# Heat Generation and Pain Assessment in Piezosurgery Versus Conventional Drilling for Implant Placement: A Systematic Review

**DOI:** 10.7759/cureus.71396

**Published:** 2024-10-13

**Authors:** Neha Jain, Pankaj Dhawan, Sapna Rani, Janvi Kalra

**Affiliations:** 1 Department of Prosthodontics, Manav Rachna Dental College, Faridabad, IND

**Keywords:** heating, implant surgery, pain on vas, piezo surgery, ultrasonic bone surgery

## Abstract

The development of new technologies in implant dentistry has led to the introduction of piezosurgery, which utilizes ultrasonic vibrations for micrometric and selective bone cutting. It is hypothesized to reduce trauma to bone tissue, thereby potentially improving the healing process compared to traditional drilling methods. This systematic review examines the differences in heat generation and pain perception between piezosurgery and conventional drilling techniques for dental implant placement. An extensive review of literature was performed using various databases, including PubMed via MEDLINE, EBSCO, and Google Scholar. Out of an initial pool of 2,279 articles, nine studies met the predefined inclusion criteria and were included in the final analysis following title and abstract screening and subsequent full-text evaluation. The quality of the selected studies was rigorously assessed using the Risk of Bias (RoB 2.0), Risk of Bias in Non-randomized Studies - of Interventions (ROBINS-I), and Quality Assessment Tool for In Vitro Studies (QUIN) tools. Data pertaining to thermal effects and postoperative pain associated with piezosurgery relative to conventional drilling protocols were collected. The results indicated a significant reduction in postoperative pain with piezosurgery; however, the technique was associated with higher intraoperative temperatures during osteotomy. These findings provide valuable insights into the potential benefits and limitations of piezosurgery in dental implant procedures.

## Introduction and background

There are multiple factors governing the long-term success of dental implants where implant site preparation technique plays a major role. To establish a stable bond between dental implants and bone tissue, the surgical site must be prepared with minimal trauma. Excessive mechanical or thermal stress can adversely affect the bone healing process. Advances in bone-cutting techniques and osteotomies, particularly in implant surgery, have focused on reducing tissue damage [1]. Ultrasonic implant site preparation has been proposed to be beneficial in this regard, especially in complex cases. Piezosurgery, which employs ultrasonic vibrations to enable micrometric and selective bone cutting, is considered a highly precise and safe technique for osteotomies [2]. Many studies have confirmed the superiority of piezosurgery over conventional drilling methods in terms of implant stability and postoperative healing. Factors such as the cutting tool’s design, drilling pressure and speed, and the use of irrigation significantly influence the degree of bone tissue damage, thermal stress, and postoperative pain [3-6].

This systematic review will compare piezosurgery and conventional drilling methods used in dental implant placement, with an emphasis on heat generation and patient-reported pain levels. Literature suggests that piezosurgery, due to its ultrasonic mechanism, may generate less mechanical stress but has the potential to increase intra-operative heat at the osteotomy site, as compared to conventional drilling [7-12]. The use of copious irrigation in piezosurgery can help mitigate this thermal effect, but proper control over the cutting tool's application remains critical. In terms of patient comfort, several studies have reported a significant reduction in postoperative pain with piezosurgery, attributed to its less invasive approach and precision in bone cutting, while others have reported minimal difference [6,13,14]. This review seeks to provide a clearer understanding of the thermal and pain-related outcomes of piezosurgery compared to conventional methods, contributing to better-informed decisions in clinical practice for dental implant procedures.

## Review

Methodology

**Figure 1:**
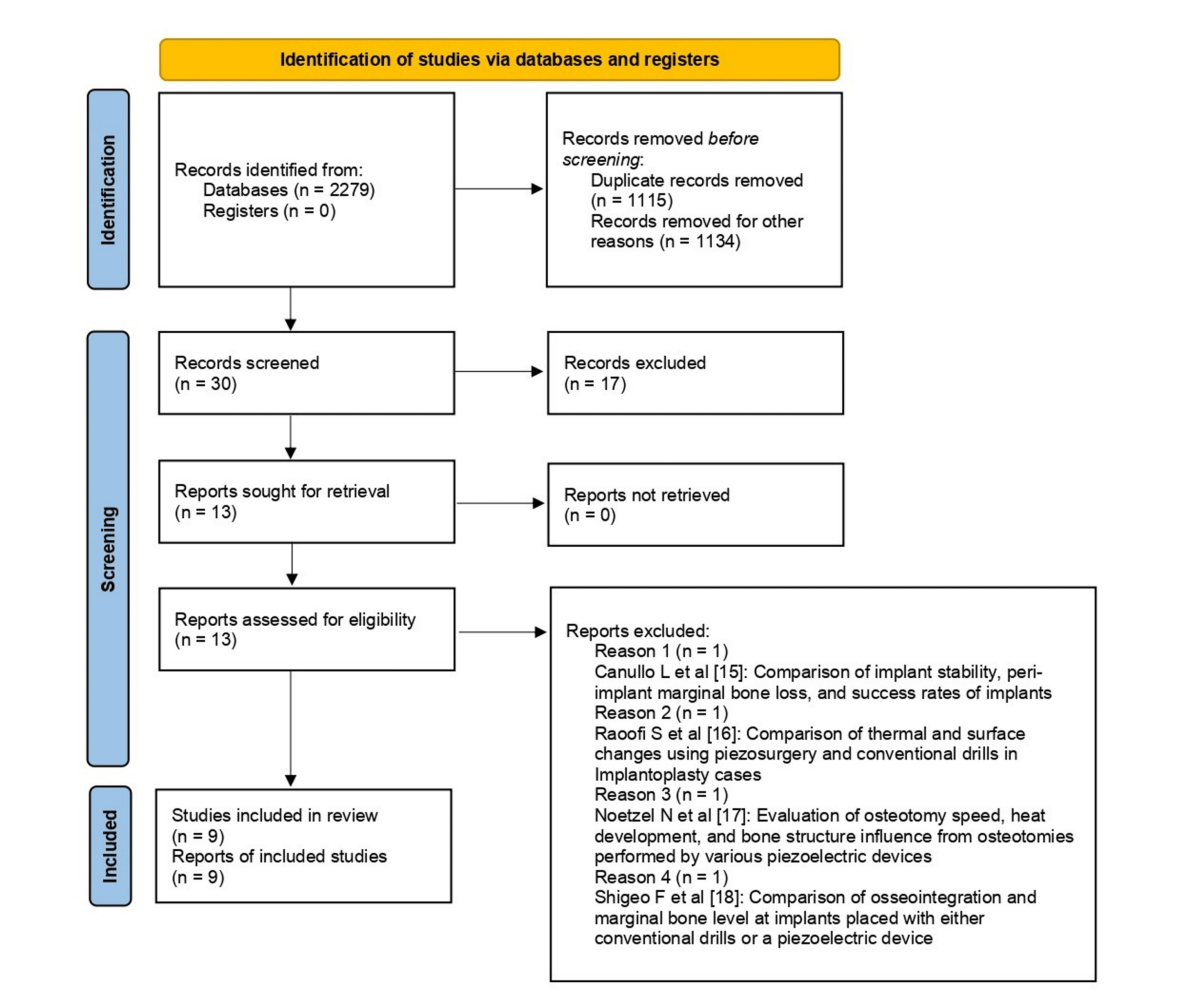
PRISMA flowchart depicting the process of inclusion of studies PRISMA, Preferred Items for Systematic Review and Meta-Analysis

The current systematic review was conducted according to the Preferred Items for Systematic Review and Meta-Analysis (PRISMA) guidelines, as depicted in Figure 1. The protocol for the review was registered on the International Prospective Register of Systematic Reviews (PROSPERO) with ID: CRD42024569302.

The following PICO (participants, intervention, comparison, outcome) question was framed to identify the relevant records for the systematic review, “What is the difference in heat generation and pain score in piezosurgery when compared to conventional drilling for implant placement?”. The population was identified as patients/bone specimens with implant placement; intervention: piezosurgery for implant site preparation; comparator/control: conventional drilling for implant site preparation; outcomes: heat generation and postoperative pain assessment.

Search Strategy

The search for relevant articles for the systematic review was conducted in the following databases: PubMed via MEDLINE, EBSCO, and Google Scholar from January 2014 to June 2024. The search strategy was modified in accordance with each database without any language restriction. The Medical Subject Headings (MeSH) terms, along with keywords, were used with appropriate Boolean operators to identify the articles. The following search strategy was devised: ((“heating” OR “heat generation” OR “pain assessment”) AND (“ultrasonic implant” OR “piezosurgery”) AND (“conventional drill” OR “conventional implant”)).

Inclusion Criteria

The inclusion criteria consist of randomized controlled trials (RCTs), non-randomized controlled trials/studies of intervention (NRSIs), in vitro experiments, ex vivo studies, and animal studies.

Exclusion Criteria

The exclusion criteria include literature reviews, case reports, systematic reviews, and studies not reporting both osteotomy techniques.

Study Selection

Two independent investigators (N.J. and J.K.) performed the research to identify relevant articles from the three databases. Manual searches were also conducted, and references and bibliographies were searched to identify relevant records. Titles and abstracts were screened following, and duplicates were removed. The remaining articles were assessed against predefined inclusion criteria. The other investigators (P.D. and S.R.) were consulted in case of any disagreement. The final decision regarding the inclusion of articles was made via discussion. Relevant data on heat generation and pain scores was extracted and reviewed. The initial search identified 2,279 articles. After excluding the articles based on titles, 36 articles were assessed on the basis of abstracts, out of which 17 were rejected, and six duplicates were removed. Thirteen articles were considered for full-text review, out of which four studies [15-18] were excluded, as detailed in Figure 1. Thus, nine studies [6-14] were reviewed with the details illustrated in Table 1.

**Table 1:** Characteristics of studies included in the review comparing piezosurgery and conventional drilling ISP, implant site preparation; NA: not applicable; PS, piezosurgical; SEM, scanning electron microscope; T, temperature; UISP, ultrasonic implant site preparation; VAS, visual analog scale

S. no.	Included study	Year	Type of study	Objective	Participant/population	Outcome		p-value
						Heat generation	Pain assessment	
1.	Stelzle F et al. [7]	2014	Ex vivo	To compare PS versus conventional drilling methods for ISP - focusing on heat generation on hard tissue and the expenditure of ISP time	Three hundred sixty ex vivo pig heads.	PS yields the highest mean temperatures (48.6 ± 3.4°C). The highest temperature recorded with the conventional drill was 45.5°C.	NA	<0.05
2.	Peker Tekdal G et al. [13]	2016	Randomized controlled trial	To evaluate the effect of PS implant osteotomy on postoperative pain levels	Thirty-eight osteotomies (19 patients) were prepared with PS and conventional drilling in the posterior maxilla in a split-mouth design.	NA	Mean VAS score for PS on day 1 = 20.3, day 2 = 12.0, day 7 = 4.5; mean VAS score for conventional drill on day 1 = 28.9, day 2 = 12.0, day 7 = 5.7	<0.05
3.	Fugito Junior K et al.[8]	2018	In vitro	To compare temperature variation during the preparation of implant surgical beds using conventional rotary implant burs versus ultrasonic tips	Sixty cortical bone samples of fresh bovine femur.	The highest recorded temperature was 38.10°C in the piezosurgery group, which did not exceed the critical value of 47°C, while the highest temperature in the conventional drill group was 33.6°C.	NA	>0.05
4.	Scarano A et al. [6]	2018	Randomized controlled trial; parallel arm	To evaluate postoperative pain measurement through VAS between piezosurgery and conventional drilling	Fifty patients received dental implants in the posterior mandible.	NA	Mean VAS score for ultrasonic device on day 1 = 12.33 ± 2.32, day 2 = 15.32 ± 3.34, and day 4 = 0.82 ± 0.01; mean VAS score for drill on day 1 = 16.33 ± 4.12, day 2 = 19.22 ± 2.22, day 4 = 40.92 ± 0.3	<0.05
5.	Lajolo C et al. [9]	2018	Ex vivo	To evaluate the apical cortical plate temperature increase in piezosurgery and conventional drill in a porcine rib ex vivo model	A total of 24 implant sites were prepared on 24 porcine ribs.	The average temperature increase was 0.07°C for group 1 (drill system 1,000 load g); 0.22°C for group 2 (drill system 1,500 g); 9.18°C for group 3 (piezosurgery 1,000 g); and 8.17°C for group 4 (piezosurgery 1,500 g).	NA	<0.05
6.	Maglione M et al. [14]	2019	Observational study	To compare the operative time, the postoperative pain, and the amount of painkillers taken by the patient during the healing period in piezosurgery and conventional drilling	Sixty-five patients were treated using a split-mouth model in maxilla or mandible.	NA	Mean VAS score for drill on day 1 = 4.05, day 2 = 3.58, day 7 = 0.33; mean VAS score for ultrasonic device on day 1 = 2.51, day 2 = 2.09, and day 7 = 0.09	<0.05
7.	Rebaudi A et al. [10]	2020	In vitro	To evaluate the cortical and cancellous bone damage procured by conventional and UISP techniques using SEM	Five bovine ribs with 25 × 4 × 1 cm dimensions.	Ultrasonic vibrations provided a gentler action involving less heat generation.	NA	Not mentioned
8.	Bhargava N et al. [11]	2023	Ex vivo	To investigate differences in heat generation at three different levels (T1, T2, T3) from the crest of porcine ribs in piezoelectric and conventional drilling	Sixty implant sites with three different levels (T1, T2, T3) in the crest of porcine ribs.	Mean temperature with piezoelectric system: T1 = 29.53, T2 = 15.98, T3 = 16.62; mean temperature with conventional drills: T1 = 22.89 ± 1.10, T2 = 23.31 ± 0.79, T3 = 23.42 ± 1.36	NA	<0.05
9.	Aquilanti L et al. [12]	2023	In vitro	To measure temperature variation generated during the initial osteotomy using both rotatory and piezo-surgical inserts	A total of 315 implant site preparations were performed in an artificial bone sample.	Highest recorded temperature in the piezosurgery group = 88.27 ± 35.77°C; highest recorded temperature in the conventional drill group = 21.63 ± 2.19°C	NA	<0.05

Data Extraction

Using a preconfigured table, the author's name, year of publication, study design, population/sample, findings, and other pertinent information were extracted from the data. A spreadsheet (Google Sheets, Alphabet Inc., Mountain View, CA) was utilized to compile the information into evidence tables.

Risk of Bias Assessment

**Figure 2:**
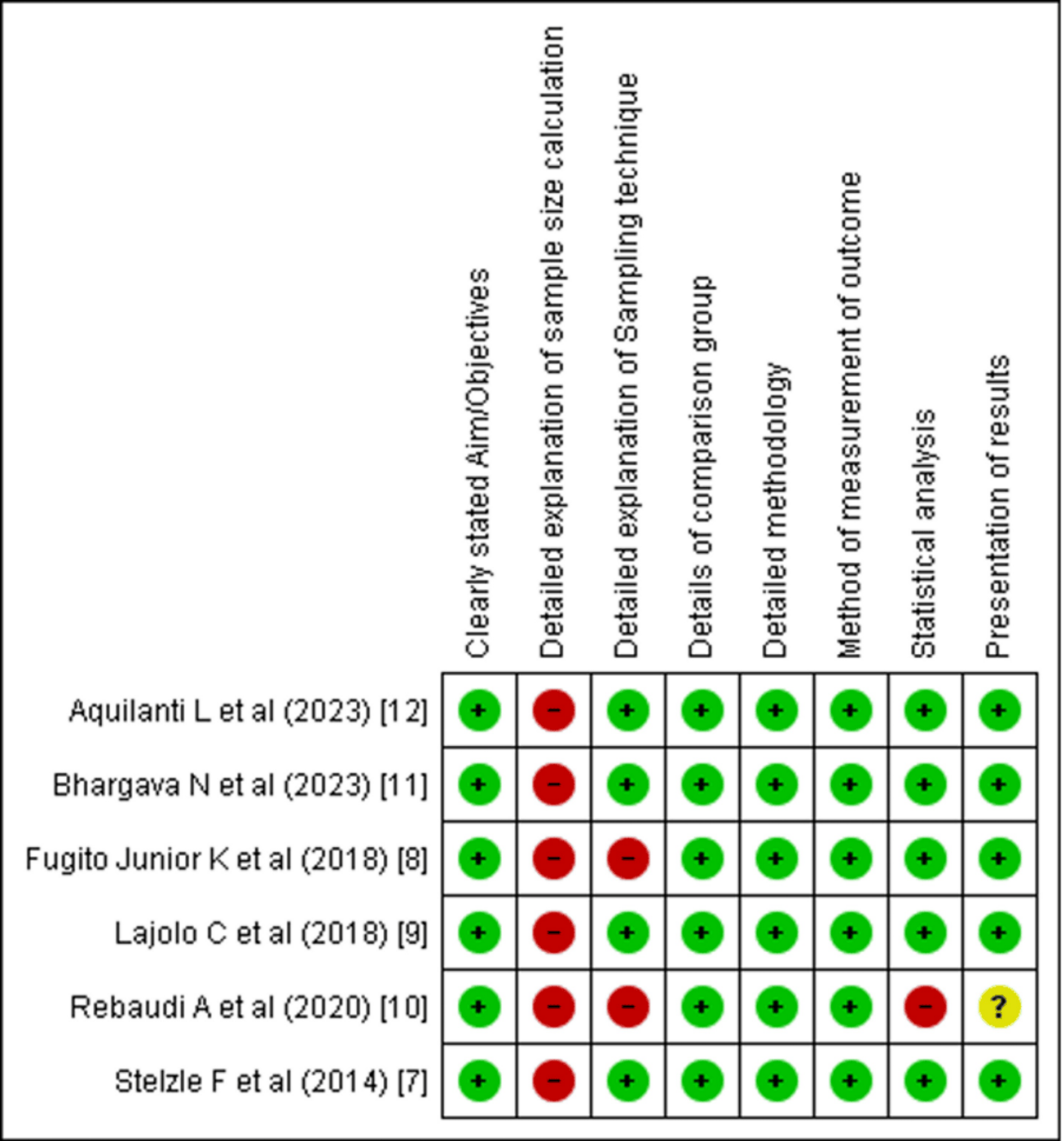
Risk of bias assessment of in vitro studies and ex vivo studies using the QUIN tool QUIN, Quality Assessment Tool for In Vitro Studies Six studies [7-12]

**Figure 3:**
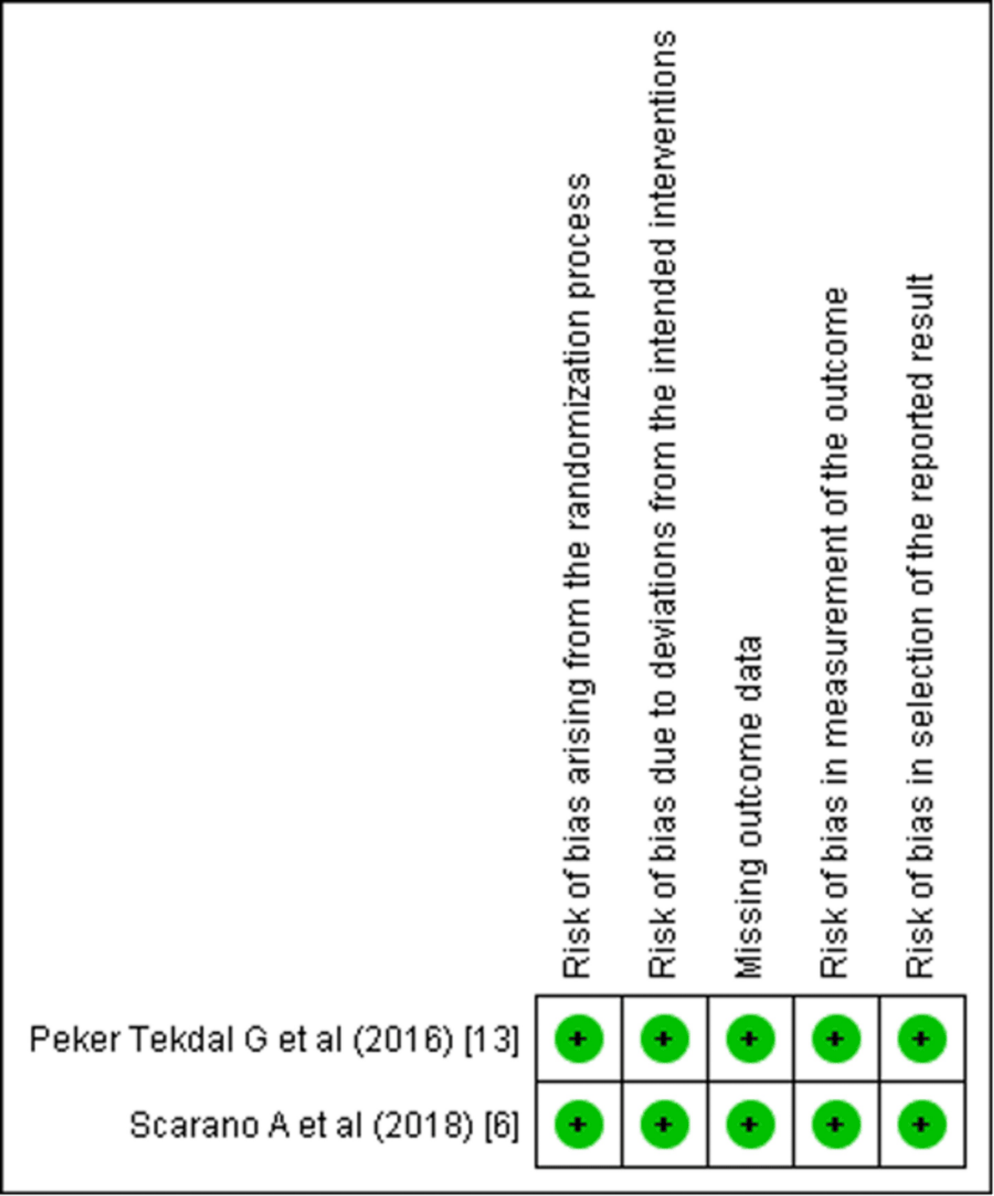
Risk of bias assessment of randomized controlled trials using RoB 2.0 tool (Cochrane) RoB, Risk of Bias Two studies [6,13]

**Figure 4:**
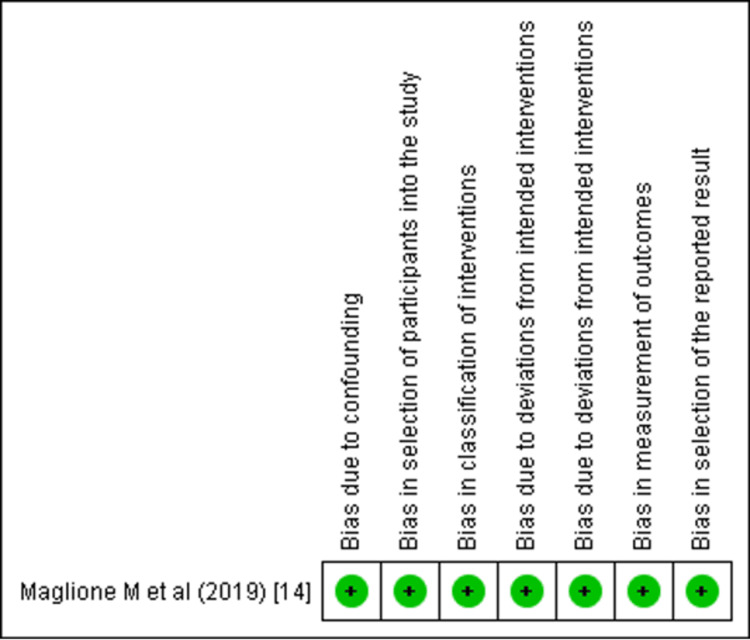
Risk of bias assessment using ROBINS-I tool (Cochrane) ROBINS-I, Risk of Bias in Non-randomized Studies - of Interventions One study [14]

Out of the selected articles, two were RCTs, one was NRSI, and the remaining six were in vitro and ex vivo studies. The Cochrane tools were used to determine the risk of bias in individual clinical trials. Risk of Bias (RoB 2.0) tool was used to determine individual risk in RCTs, Risk of Bias in Non-randomized Studies - of Interventions (ROBINS-I) was used for the NRSIs, and the Quality Assessment Tool for In Vitro Studies (QUIN) tool was used to determine the risk in in vitro and ex vivo studies (Supplementary Materials 1-3) [19-21]. The studies were categorized as high, moderate, or low risk. The QUIN tool assessed the studies on eight criteria that were applicable as per the requisites of this review. These included the sampling strategy, sample size calculation, comparison group information, methodology, outcome measuring method, statistical analysis, and results presentation, all of which must be clearly stated. Out of the six in vitro and ex vivo studies, five showed low risk, and one showed a moderate risk (Figure 2). Both the RCTs and NRSIs exhibited a low risk of bias (Figure 3 and Figure 4).

Discussion

Piezosurgery is gaining popularity in implantology for implant site preparation due to its selective cut, cavitational effect, and preservation of soft tissues [22]. It provides a more accurate implant positioning and preservation of bone vitality when compared to conventional osteotomy preparation [14]. The objective of this systematic review was to compare this less invasive osteotomy method to conventional drilling in relation to heat generation and pain assessment in terms of visual analog scale (VAS) scores. Nine studies were included to assess these parameters, out of which six studies [7-12] reported on heat generation and three studies [6,13,14] reported on postoperative pain levels. Out of the nine included studies, eight had a low risk of bias [6-9,11-14], while one study had a moderate risk of bias [10]. The study conducted by Rebaudi A et al. [10] had poorly defined objectives and presentation of results.

Heat Generation

The comparison of heat generation between piezosurgery and conventional drilling in dental implant placement, as derived from various studies, shows significant variability in temperature outcomes across both techniques. Five of the included studies [7-9,11,12] reported that piezosurgery tends to generate higher temperatures than conventional drilling under certain conditions. Meanwhile, one study showed lesser heat generation in piezosurgery than in conventional drilling [10]. Stelzle F et al. [7] recorded a higher mean temperature for piezosurgery (48.6°C) compared to conventional drilling (45.5°C). Another study by Fugito Junior K et al. [8] showed a lower peak temperature in the piezosurgery group (38.1°C), staying below the critical 47°C threshold, while the conventional drill recorded an even lower temperature (33.6°C). In terms of temperature increase under load, piezosurgery consistently produced greater heat compared to conventional drilling, with temperature rise of 9.18°C and 8.17°C, versus minimal increases (0.07°C and 0.22°C) for the drill group [9]. Bhargava N et al. [11] and Aquilanti L et al. [12] noted extreme heat spikes in piezosurgery, 29.53°C and 88.27°C, respectively, highlighting the potential for higher temperatures if not managed carefully. However, only one of the included studies by Rebaudi A et al. [10] noted that piezosurgery, due to its ultrasonic vibrations, generated less heat overall, suggesting technique and irrigation play roles in temperature control.

Lamazza L et al. [23] identified three key factors influencing temperature rise, which include working load, management of handpiece movements, and bone characteristics. Piezosurgery tips produced the most significant heat due to external irrigation and the initial resistance of the cortical bone. The study highlighted the importance of controlling hand movements and applied load to limit temperature elevation. Möhlhenrich SC et al. [24] reported that piezosurgery produces higher temperatures when there is poor irrigation and is only safe when sufficient external cooling is provided.

Postoperative Pain Assessment

The comparison of postoperative pain between piezosurgery and conventional drilling in dental implant placement, as assessed using the VAS scores, shows that piezosurgery generally results in lower pain scores, contributing to greater patient comfort [6,13,14]. In the study by Peker Tekdal G et al. [13], the mean VAS scores for piezosurgery were consistently lower for conventional drilling on day 1 (20.3 versus 28.9) and day 7 (4.5 versus 5.7), with both methods showing similar scores on day 2 (12.0). Scarano A et al. [6] further emphasized this trend, reporting that piezosurgery (12.33 ± 2.32 on day 1, 0.82 ± 0.01 on day 4) resulted in significantly less pain compared to conventional drilling (16.33 ± 4.12 on day 1, 40.92 ± 0.3 on day 4). Similarly, Maglione M et al. [14] observed lower VAS scores for piezosurgery (2.51 on day 1, 0.09 on day 7) compared to conventional drilling (4.05 on day 1, 0.33 on day 7). These consistent findings suggest that piezosurgery, by reducing postoperative pain, also enhances overall patient comfort during the recovery process, making it a more favorable option for patient satisfaction in implant placement. The selective and micrometric cutting ability of piezoelectric osteotomy contributes to favorable clinical outcomes, including reduced postoperative facial swelling and trismus, as well as a less traumatic experience for the patient [25-27]. Overall, while piezosurgery reduces postoperative pain, it, however, generates more heat compared to conventional drilling, making temperature regulation a critical factor in its application.

The present study had certain limitations. There were only three in vivo studies included, out of which merely two were RCTs. Also, the data provided in the in vitro studies reporting on heat generation were heterogeneous in terms of the type of sample, methodology, and measurement of outcomes, because of which meta-analysis was not possible. There was an unavailability of quantitative data in some of the studies comparing the temperature variations between piezosurgery and conventional drilling. Further reviews should focus on including more RCTs with homogenous data to conduct a meta-analysis. Authors should report on quantitative data to reduce bias and strengthen the evidence.

## Conclusions

The following conclusions can be drawn from this systematic review: (1) Heat generation is higher in osteotomy sites prepared using piezosurgery than in conventional drills. This is attributed to the lower cutting efficiency of piezoelectric burs, which leads to prolonged operation times and, consequently, more heat buildup. (2) Despite the increased head generation, patients undergoing implant placement using piezosurgery experienced greater comfort and lower pain levels compared to conventional drilling. This is due to the atraumatic nature of piezosurgery, which results in reduced soft tissue trauma and better overall postoperative outcomes. (3) The reduced soft tissue trauma and lower postoperative pain levels lead to a faster and smoother postoperative recovery.

## Appendices

**Table 2:** Risk of bias assessment of in vitro studies using QUIN tool QUIN, Quality Assessment Tool for In Vitro Studies

Parameters	Included studies					
	Stelzle F et al. [7]	Fugito Junior K et al. [8]	Lajolo C et al. [9]	Rebaudi A et al. [10]	Bhargava N et al. [11]	Aquilanti L et al. [12]
Clearly stated aim/objectives	2	2	2	2	2	2
Detailed explanation of sample size calculation	0	0	0	0	0	0
Detailed explanation of Sampling technique	2	0	2	0	2	2
Details of comparison group	2	2	2	2	2	2
Detailed methodology	2	2	2	2	2	2
Operator details	0	0	0	0	0	0
Randomization	0	0	2	0	0	0
Method of measurement of outcome	2	2	2	2	2	2
Operator assessor details	0	0	0	0	0	0
Blinding	0	0	0	0	0	0
Statistical analysis	2	2	2	0	2	2
Presentation of results	2	2	2	1	2	2
Total score	14	12	14	9	14	14
Bias evaluation (score × 100 / 2 × number of criteria applicable)	87.5% (low risk of bias)	75% (low risk of bias)	87.5% (low risk of bias)	56.25% (moderate risk of bias)	87.5% (low risk of bias)	87.5% (low risk of bias)

**Table 3:** Risk of bias assessment of randomized controlled trials using RoB 2.0 rool (Cochrane) RoB, Risk of Bias

Parameter/domain	Included study	
	Scarano A et al. [6]	Tekdal G et al. [13]
1. Risk of bias arising from the randomization process		
1.1 Was the allocation sequence random?	Yes	Yes
1.2 Was the allocation sequence concealed until participants were enrolled and assigned to interventions?	Yes	Yes
1.3 Did baseline differences between intervention groups suggest a problem with the randomization process?	No	No
Risk-of-bias judgement	Low	Low
2. Risk of bias due to deviations from the intended interventions		
2.1. Were participants aware of their assigned intervention during the trial?	No	No
2.2. Were carers and people delivering the interventions aware of participants' assigned intervention during the trial?	Yes	Yes
2.3. If Y/PY/NI to 2.1 or 2.2: Were there deviations from the intended intervention that arose because of the trial context?	No	No
2.4 If Y/PY to 2.3: Were these deviations likely to have affected the outcome?	-	-
2.5. If Y/PY/NI to 2.4: Were these deviations from intended intervention balanced between groups?	-	-
2.6 Was an appropriate analysis used to estimate the effect of assignment to intervention?	Yes	Yes
2.7 If N/PN/NI to 2.6: Was there potential for a substantial impact (on the result) of the failure to analyse participants in the group to which they were randomized?	-	-
Risk-of-bias judgement	Low	Low
3. Missing outcome data		
3.1 Were data for this outcome available for all, or nearly all, participants randomized?	Yes	Yes
3.2 If N/PN/NI to 3.1: Is there evidence that the result was not biased by missing outcome data?	-	-
3.3 If N/PN to 3.2: Could missingness in the outcome depend on its true value?	-	-
3.4 If Y/PY/NI to 3.3: Is it likely that missingness in the outcome depended on its true value?	-	-
Risk-of-bias judgement	Low	Low
4: Risk of bias in measurement of the outcome		
4.1 Was the method of measuring the outcome inappropriate?	Yes	Yes
4.2 Could measurement or ascertainment of the outcome have differed between intervention groups?	No	No
4.3 If N/PN/NI to 4.1 and 4.2: Were outcome assessors aware of the intervention received by study participants?	Yes	No
4.4 If Y/PY/NI to 4.3: Could assessment of the outcome have been influenced by knowledge of intervention received?	No	-
4.5 If Y/PY/NI to 4.4: Is it likely that assessment of the outcome was influenced by knowledge of intervention received?	-	-
Risk-of-bias judgement	Low	Low
5: Risk of bias in selection of the reported result		
5.1 Were the data that produced this result analysed in accordance with a pre-specified analysis plan that was finalized before unblinded outcome data were available for analysis? Is the numerical result being assessed likely to have been selected, on the basis of the results, from...	Yes	Yes
5.2. ... multiple eligible outcome measurements (e.g. scales, definitions, time points) within the outcome domain?	No	Yes
5.3 ... multiple eligible analyses of the data?	No	No
Risk-of-bias judgement	Low	Low
Overall risk of bias	Low	Low

**Table 4:** Risk of bias assessment using ROBINS-I tool (Cochrane) ROBINS-I, Risk of Bias in Non-randomized Studies - of Interventions

Included study	Parameter/domain	Comment
Maglione M et al. [14]	1. Bias due to confounding	
	1.1 Is there potential for confounding of the effect of intervention in this study?	No
	If N/PN to 1.1: the study can be considered to be at low risk of bias due to confounding and no further signalling questions need to be considered	
	If Y/PY to 1.1: determine whether there is a need to assess time-varying confounding:	
	1.2. Was the analysis based on splitting participants’ follow-up time according to intervention received?	-
	If N/PN, answer questions relating to baseline confounding (1.4 to 1.6) If Y/PY, go to question 1.3.	
	1.3. Were intervention discontinuations or switches likely to be related to factors that are prognostic for the outcome?	-
	If N/PN, answer questions relating to baseline confounding (1.4 to 1.6) If Y/PY, answer questions relating to both baseline and time-varying confounding (1.7 and 1.8)	
	1.4. Did the authors use an appropriate analysis method that controlled for all the important confounding domains?	-
	1.5. If Y/PY to 1.4: Were confounding domains that were controlled for measured validly and reliably by the variables available in this study?	
	1.6. Did the authors control for any post-intervention variables that could have been affected by the intervention?	-
	1.7. Did the authors use an appropriate analysis method that controlled for all the important confounding domains and for time-varying confounding?	-
	1.8. If Y/PY to 1.7: Were confounding domains that were controlled for measured validly and reliably by the variables available in this study?	-
	Risk of bias judgement	Low
	2. Bias in selection of participants into the study	
	2.1. Was selection of participants into the study (or into the analysis) based on participant characteristics observed after the start of intervention?	No
	If N/PN to 2.1: go to 2.4	
	2.2. If Y/PY to 2.1: Were the post-intervention variables that influenced selection likely to be associated with intervention?	-
	2.3 If Y/PY to 2.2: Were the post-intervention variables that influenced selection likely to be influenced by the outcome or a cause of the outcome?	-
	2.4. Do start of follow-up and start of intervention coincide for most participants?	Yes
	2.5. If Y/PY to 2.2 and 2.3, or N/PN to 2.4: Were adjustment techniques used that are likely to correct for the presence of selection biases?	-
	Risk of bias judgement	Low
	3. Bias in classification of interventions	
	3.1 Were intervention groups clearly defined?	Yes
	3.2 Was the information used to define intervention groups recorded at the start of the intervention?	Yes
	3.3 Could classification of intervention status have been affected by knowledge of the outcome or risk of the outcome?	No
	Risk of bias judgement	Low
	4. Bias due to deviations from intended interventions	
	If your aim for this study is to assess the effect of assignment to intervention, answer questions 4.1 and 4.2	
	4.1. Were there deviations from the intended intervention beyond what would be expected in usual practice?	No
	4.2. If Y/PY to 4.1: Were these deviations from intended intervention unbalanced between groups and likely to have affected the outcome?	-
	If your aim for this study is to assess the effect of starting and adhering to intervention, answer questions 4.3 to 4.6	
	4.3. Were important co-interventions balanced across intervention groups?	Yes
	4.4. Was the intervention implemented successfully for most participants?	Yes
	4.5. Did study participants adhere to the assigned intervention regimen?	Yes
	Risk of bias judgement	Low
	5. Bias due to deviations from intended interventions	
	5.1 Were outcome data available for all, or nearly all, participants?	Yes
	5.2 Were participants excluded due to missing data on intervention status?	No
	5.3 Were participants excluded due to missing data on other variables needed for the analysis?	No
	5.4 If PN/N to 5.1, or Y/PY to 5.2 or 5.3: Are the proportion of participants and reasons for missing data similar across interventions? 5.5 If PN/N to 5.1, or Y/PY to 5.2 or 5.3: Is there evidence that results were robust to the presence of missing data?	-
	Risk of bias judgement	Low
	6. Bias in measurement of outcomes	
	6.1 Could the outcome measure have been influenced by knowledge of the intervention received?	No
	6.2 Were outcome assessors aware of the intervention received by study participants?	Yes
	6.3 Were the methods of outcome assessment comparable across intervention groups?	Yes
	6.4 Were any systematic errors in measurement of the outcome related to intervention received?	No
	Risk of bias judgement	Low
	7. Bias in selection of the reported result. Is the reported effect estimate likely to be selected, on the basis of the results, from...	
	7.1. ... multiple outcome measurements within the outcome domain?	No
	7.2 ... multiple analyses of the intervention-outcome relationship?	No
	7.3 ... different subgroups?	No
	Risk of bias judgement	Low
	Overall risk of bias	Low
